# Erector Spinae Plane Block (ESPB) as an Alternative for Celiac Plexus Blocks: Expanding ESPB Indications for Mesenteric Ischemia Relief

**DOI:** 10.7759/cureus.39860

**Published:** 2023-06-02

**Authors:** Ahmed Abdelhamid, Mohmad Salim

**Affiliations:** 1 Anesthesia, Walsall Healthcare NHS Trust, Walsall, GBR

**Keywords:** celiac plexus block, ultrasound, mesenteric ischemia, erector spinae plane block, abdominal pain

## Abstract

Mesenteric ischemia is a severe condition associated with abdominal pain which often requires multimodal analgesia, including opioids or sympathetic blocks such as celiac plexus blocks. The erector spinae plane (ESPB) has emerged as a potentially effective alternative for managing pain in various surgical and non-surgical conditions. This case report explores the use of ultrasound-guided ESPB as a novel approach to pain management in a patient with acute on chronic mesenteric ischemia. A 70-year-old male with a history of mesenteric ischemia and multiple comorbidities presented with worsening diffuse abdominal pain. Despite medical and surgical treatment, the patient required a high dose of opioids for pain control. Bilateral ESPBs with continuous infusions were performed at the T6 level under ultrasound guidance. The patient reported immediate and complete relief from abdominal pain following the block, with a significant drop in the pain score. The use of opioids was significantly reduced. This case report demonstrates the potential benefits of ultrasound-guided ESPB as an alternative to traditional pain management techniques in patients with mesenteric ischemia. ESPB may provide safe, simple, and effective analgesia, reducing the need for high-dose opioids and their associated side effects. Further studies are warranted to validate these findings and explore the broader application of ESPB in the management of mesenteric ischemia pain.

## Introduction

Mesenteric ischemia occurs when the blood supply fails to adequately satisfy the metabolic needs of the internal organs [1]. Abdominal pain is the most typical symptom of mesenteric ischemia [2]. Pain intensity and severity of the ischemia are determined by the degree of stenosis and blood flow to the organs [1].

Patients suffering from mesenteric ischemia's abdominal pain often necessitate multimodal analgesia approaches, which typically involve the use of systemic analgesics such as opioids, regional techniques like epidural analgesia, or sympathetic blocks, including celiac plexus blocks or neurolysis.

Erector spinae plane block (ESPB) was first introduced by Forero et al. in 2016 as an interfascial plane block for both chronic and postsurgical or post-traumatic pain [3]. The indications for ESPB are expanding to include various surgical and non-surgical conditions.

In this case, we report the use of ultrasound-guided ESPB as an alternative to celiac plexus block for pain management in a patient diagnosed with acute on chronic mesenteric ischemia who is unfit for vascular surgical intervention. Our aim for this case is to contribute to the growing literature that demonstrates the safety, simplicity, and effectiveness of ESPB in managing thoracoabdominal pain.

## Case presentation

**Figure 1:**
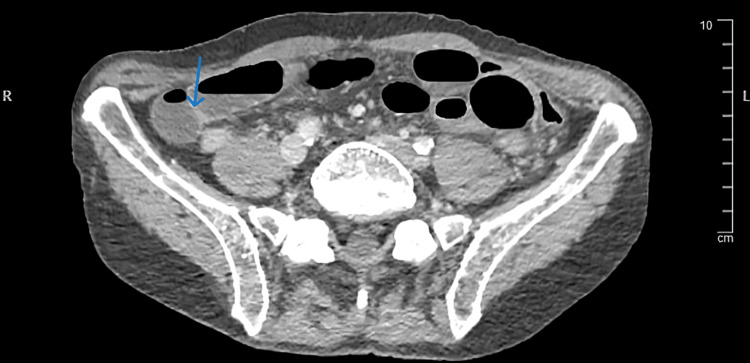
Ileal loop suspicious of bowel ischemia

**Figure 2:**
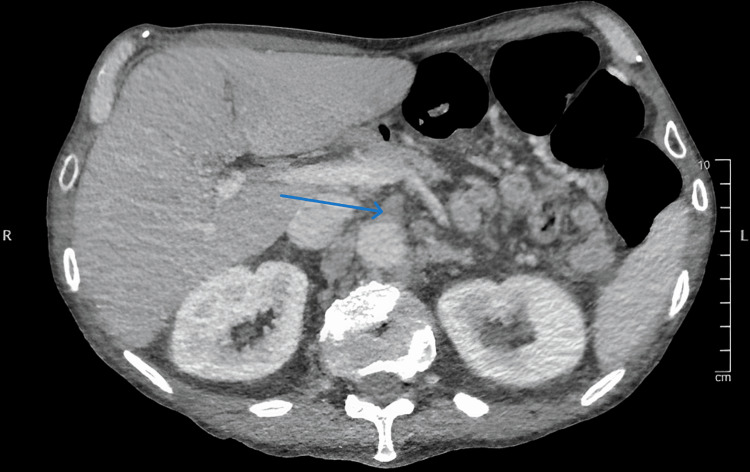
Celiac trunk occlusion

**Figure 3:**
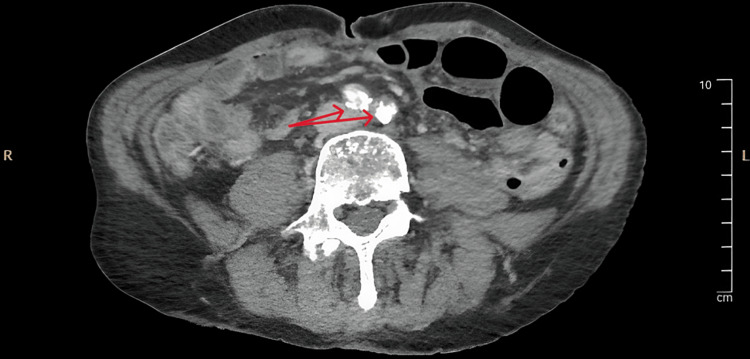
Common iliac artery occlusion

A 70-year-old male was admitted to the surgical ward with worsening diffuse abdominal pain. A few days prior to his admission patient was discharged home from vascular surgery after he was medically treated for chronic mesenteric ischemia and was started on Aspirin and Atorvastatin. His medical co-morbidities were hypertension, ischemic heart disease, chronic obstructive pulmonary disease, peptic ulcer disease with a previously surgically treated duodenal perforation, and depression. The patient was an ex-heavy smoker and lived independently. An urgent CT abdomen and pelvis with a contrast scan showed a small bowel-ileal loop which was suspicious of developing bowel ischemia (Figure 1). The scan also showed an occlusion and absent opacification at the origin of the celiac trunk and proximal superior mesenteric artery (SMA) (Figure 2). There was also an occlusion of the left common iliac artery and a poorly opacified external iliac branch (Figure 3). An emergency organ-saving laparotomy was performed overnight in an attempt to salvage viable bowel. However, due to the poor state of the vascular bed, vascular intervention (such as embolectomy) was not performed to fix the occlusion leading to ischemia. The surgery resulted in the resection of a segment of the small bowel and a formation of an ileostomy. Both the surgeon and anesthetist predicted that the patient would continue to suffer chronically from ischemic pain, hence the need for more potent and longer postoperative analgesia. ESPB and celiac blocks were suitable options. However, we believed ESPBs carry a lower risk of complications and are considered to be less technically demanding than celiac blocks.

Prior to surgery, the patient was requiring higher doses of sustained release oral morphine (60mg), codeine (240mg) with poor control of his pain. Patient scored between 2 and 3 on pain VAS (Visual analogue scale) of 0 to 3. Postoperatively, morphine PCA was initiated and in the first 24 hours patient required 46mg of immediate-release morphine. This indicated a referral to the local acute pain services for consideration of appropriate regional block. It is worth mentioning that the patient was feeling drowsy, and his inflammatory markers started rising despite medical management. ESPB was an intriguing option to consider. After discussion with patient and obtaining an informed consent, bilateral ESPBs were performed at the level of T6 under ultrasound guidance.

Using a high-frequency linear probe which was placed longitudinally few centimeters from the midline; the transverse process of T6 was identified as well as trapezius and erector spinae muscles. A 16G Tuohy needle was inserted after local skin infiltration. The tip of the needle touched the transverse process and hydrodissection using saline was done to confirm fascial plane. Initially 20mL of 0.2% ropivacaine was injected followed by bilateral catheters insertion. Real-time ultrasound confirmed excellent cephalic and caudal spread of the local anesthetic with no intravascular uptake. Patient reported immediate complete relief for his abdominal pain and his numerical pain score dropped dramatically to 0/3. Infusion of ropivacaine 0.2% 0-10 mL/hr was initiated. Patient was reviewed regularly by acute pain services. ESPB infusions carried on for seven days with no additional opioid requirements until the patient was commenced on fentanyl patches.

## Discussion

Patients with mesenteric ischemia pain present as either acute or chronic or most commonly acute on chronic. Acute mesenteric ischemia is associated with sudden interruption of the blood supply to a segment of the small intestine usually secondary to thrombosis of a previously stenotic mesenteric vessel and has an overall mortality of 60%-80% [1]. More than 90% of chronic mesenteric ischemia cases can be attributed to plaque build-up due to atherosclerotic disease. Patients with mesenteric ischemia should undergo revascularization surgery. Medical treatment is usually reserved for patients who are not fit for vascular surgery [1].

Acute mesenteric ischemia’s most typical presentation is moderate to severe diffuse visceral non-localized abdominal pain that is disproportionate to examination findings. In some cases, the pain does not respond to opioid analgesia [4,5]. On the other hand, chronic ischemia patients present with postprandial abdominal pain, nausea, or vomiting, and their pain is usually proportionate to the level of stenosis of the vessel [1]. Management of chronic or inoperable acute mesenteric ischemia abdominal pain can be complex and difficult and often require the administration of a high dose of opioids, which are associated with several side effects.

While opioids are commonly used in visceral abdominal pain, there is limited research on their use in mesenteric ischemia. Opioid side effects are common and well-known. In the gastrointestinal tract, opioids inhibit acetylcholine release, leading to reduced bowel motility and inducing postoperative paralytic ileus or causing opioid-induced constipation [6,7]. Continuous opioid treatment could affect abdominal perfusion or mask ongoing ischemia leading to delayed diagnosis [7]. However, in this patient, the diagnosis of inoperable mesenteric ischemia was already established, so our priority was to control the patient's symptoms and improve the quality of life. And that’s why we needed multimodal analgesia.

Upper abdominal pain impulses are carried by afferent nociceptive nerve fibers (such as the splanchnic nerve) that relay in the celiac plexus to make their way to the central nervous system [8]. And an effective approach to relieving the pain is to block these signals at the celiac plexus or splanchnic nerve levels [9].

Kappis et al. in 1914 first introduced celiac plexus block using local anesthetic and since then various techniques have been developed using radiology [10]. Imaging-guided celiac plexus blocks are now playing an important role in managing abdominal pain. Celiac plexus blocks are often used to determine if the pain is sympathetically mediated via the celiac plexus and to assess if the pain will be managed with a more permanent and invasive neurolysis [9]. It is worth mentioning that both procedures are technically demanding apart from being contraindicated in coagulopathic patients or those with intraabdominal infection or sepsis [9,11,12].

Celiac plexus block generally carries a higher risk of complications due to its proximity to major blood vessels such as the aorta or the inferior vena cava which can lead to significant bleeding and hematoma formation [13]. It is also important to consider the complications associated with phenol use in celiac plexus blocks which include local tissue damage, neural injury, inadvertent spread to unintended areas, systemic toxicity, infection, and potential allergic reactions [14].

ESPB works by guiding the needle toward the tip of the transverse process deep to the erector spinae muscles, which are three distinct muscles that run parallel to each other from the base of the spine in the sacral region to the base of the skull. The local anesthetic is injected into the fascial plane between the erector spinae muscle and the transverse process of a vertebra, typically at the level of the thoracic vertebrae (T5 or T6). This placement ensures that the local anesthetic spread both cephalad and caudad, covering multiple spinal nerve levels. The local anesthetic also infiltrates the paravertebral space, where it can block the ventral and dorsal rami of the spinal nerves, as well as the rami communicants, which contain sympathetic fibers. As a result, the ESPB provides both somatic and visceral pain relief. The ESPB in this case needs to be performed bilaterally to cover the ischemic bowel [15,16].

## Conclusions

This case report demonstrates the potential benefits of ultrasound-guided ESPB as an alternative to traditional pain management techniques in patients with mesenteric ischemia. ESPB may provide safe, simple, and effective analgesia, reducing the need for high-dose opioids and their associated side effects. Further studies are warranted to validate these findings and explore the broader application of ESPB in the management of mesenteric ischemia pain.
